# Evaluation of Two-Diabetes Related microRNAs Suitability as Earlier Blood Biomarkers for Detecting Prediabetes and type 2 Diabetes Mellitus

**DOI:** 10.3390/jcm7020012

**Published:** 2018-01-26

**Authors:** Haifa Abdullah Al-Muhtaresh, Ghada Al-Kafaji

**Affiliations:** Department of Molecular Medicine, College of Medicine and Medical Sciences and Al-Jawhara Centre for Molecular Medicine, Genetics and Inherited Disorders, Arabian Gulf University, Manama, Kingdom of Bahrain; haifa.abdullah@gmail.com

**Keywords:** prediabetes, type 2 diabetes, blood microRNAs, miR-375, miR-9, biomarkers

## Abstract

Increased the incidence of prediabetes and type 2 diabetes (T2D) worldwide raises an urgent need to develop effective tools for early disease detection to facilitate future preventive interventions and improve patient’s care. We evaluated the suitability of diabetes-related miR-375 and miR-9 as earlier biomarkers for detecting prediabetes and T2D.TaqMan-based RT-qPCR was used to quantify the expression of miRNAs in peripheral blood of 30 prediabetes patients, 30 T2D patients and 30 non-diabetic healthy controls. Compared to controls, miR-375 and miR-9 were expressed at higher levels in prediabetes patients and progressively more enriched in T2D patients. Both miRNAs were directly associated with the presence of prediabetes and T2D independently of known risk factors to T2D and miR-375 was independently associated with the development of T2D. Both miRNAs were positively correlated with the glycemic status and other T2D risk factors. The ROC analysis indicated good diagnostic abilities for miR-375 to distinguish overall patients from control and prediabetes from T2D patients. Whereas, miR-9 showed lower values and borderline significance in discriminating the subject groups. The combination of miRNAs enhanced the predictability to discriminate patients from control. These results suggest that miR-375 and miR-9 are associated with the susceptibility to developing T2D and miR-375 alone or in combination with miR-9 could serve as biomarkers for early detection of prediabetes and T2D.

## 1. Introduction

In parallel with increasing the prevalence of type 2 diabetes (T2D) globally, the incidence of prediabetes has increased substantially and has been estimated to affect 482 million people by 2040 [1,2]. T2D is a chronic metabolic disease characterized by increased blood glucose levels (hyperglycemia) as a result of inadequate insulin secretion due to pancreatic beta (β)–cell dysfunction and insulin resistance [3,4]. The pathogenesis of T2D is complex and involves an interplay between genetic, epigenetic and environmental factors [5]. At first insulin resistance is accompanied by a compensatory increase in insulin secretion by the β–cells and this could last several years. Subsequently, β–cells fail to compensate for increased insulin resistance and then hyperglycemia develops [6]. Prolonged hyperglycemia is associated with vascular complications, which are the most serious manifestations of the disease and decrease the individual’s life expectancy [7].

Prediabetes is an intermediate state of hyperglycemia or state of blood glucose levels above normal but below diabetes thresholds. It is typically defined by impaired fasting glucose (IFG) and/or impaired glucose tolerance (IGT). The primary defects observed in T2D that are impaired function of β–cells and insulin resistance can be detected in both subjects with IGT and IFG [8,9]. This transition form of early metabolic abnormalities may precede T2D for years [8,9] and people with prediabetes are at increased risk of progressing to clinical T2D [10]. Indeed, 40–50% of people with IGT will progress to T2D [10]. Furthermore, prediabetes has been shown to be associated with an increased risk of cardiovascular disease [11].

Presently, there are no accepted biomarkers for early detection of T2D and the common methods used for diagnosis such as fasting glucose and glycated hemoglobin (HbA1c) are neither predictive nor permit the early detection of the disease [12,13,14]. Considering that lifestyle modifications could delay and even prevent the progression to T2D in high-risk subjects [15,16], biomarkers for early detection of prediabetes and T2D are therefore important in future preventive interventions, which would greatly improve the care of these patients.

Recently, microRNAs (miRNAs) have been shown to control gene expression post-transcriptionally through binding to complementarily to 3′UTR of the target mRNAs and repress protein production [17,18]. The gene regulatory natures of miRNAs enable them to control several important cellular processes and their dysregulation may affect major signaling pathways [19]. In T2D, dysregulated miRNAs disrupt insulin signaling pathway and various physiological processes that lead to the development and progression of the disease [20].

Among the miRNAs that play role in insulin signaling, the islet-specific miR-375 and miR-9 [21] have been demonstrated as mechanistic regulators of insulin secretion and play critical roles in glucose homeostasis and the pathogenesis diabetes [22,23].

miRNAs can be released from cells and stably present in a cell-free form in blood circulation [24] and thus represent a novel class of biomarkers. Indeed, circulating miRNAs have been shown as diagnostic, prognostic or predictive biomarkers for cancer, cardiovascular disease and other diseases [25,26,27]. Our recent studies and other studies have also highlighted the stability of miRNAs in peripheral blood and their potential use as non-invasive biomarkers for T2D [28,29].

In the present study, we aimed to evaluate the suitability of the diabetes-related miR-375 and miR-9, which are important regulator of insulin secretion, as earlier blood biomarkers for detecting prediabetes and T2D. For this purpose, we performed a TaqMan-based quantitative reverse transcription PCR (RT-qPCR) to examine the expression of the two diabetes-related miRNAs in peripheral blood of prediabetes patients, T2D patients and non-diabetic healthy individuals.

## 2. Methods

### 2.1. Study Design and Subjects

The study was conducted in accordance with the appropriate clinical and experimental ethical guidelines and was approved by the Medical Research and Ethics Committee at the College of Medicine and Medical Sciences, Arabian Gulf University, Kingdom of Bahrain. Written informed consents were obtained from the subjects before their participation.

A total of 90 subjects including 30 prediabetes patients, 30 T2D patients and 30 non-diabetic healthy individuals were enrolled in this study. Cases and controls were recruited from King Abdullah University Medical City in the Kingdom of Bahrain. All participants in the three groups were Bahraini and expected to have same genetic background.

Prediabetes and T2D were diagnosed according to the World Health Organization guidelines [30]. Prediabetes was defined as impaired fasting glucose (IFG) with FG levels ranging from 5.6 to <7.0 mmol/L, or impaired glucose tolerance (IGT) with 2-h glucose levels ranging from 7.8 to <11.1 mmol/L after a 75-g glucose load on the oral glucose tolerance test (OGTT) and glycated hemoglobin (HbA1c) levels ranging from 5.7% to 6.4%. T2D was defined as FG levels ≥7.0 mmol/L, or a 2-h OGT levels ≥11.1 mmol/L and HbA1c levels >6.5%. Type 1 diabetes (T1D) patients with onset before age of 35, with insulin therapy within 6 months of diagnosis were excluded from the study Non-diabetic healthy subjects were with no previous history of T2D with FG levels ranging from 4.8 to 5.2 mmol/L, or 2-h OGT levels <7.8 and HbA1c levels <5.7%. Subjects suffer from pancreatic diseases or autoimmune diseases as well as diabetic complications, malignant tumors or other chronic diseases which could affect the miRNA expression levels were excluded from the present study. As treatment may influence the levels of circulating miRNAs, only blood samples from patients who received no treatment at the time of the study were used.

### 2.2. Blood Collection

From each participant, peripheral whole blood (5 mL) was collected in ethylenedeminetetracetic acid (EDTA) tubes (BD Biosciences, Franklin Lakes, NJ, USA). Aliquots (0.5 mL each) of EDTA-blood were mixed with 1.3 mL RNA later (Ambion Life Technologies, Carlsbad, CA, USA) immediately after blood drawing and the tubes were stored at −80 °C until analysis.

### 2.3. RNA Extraction and Reverse Transcription

Total RNA, including miRNA, was isolated from whole blood samples using the blood miRNeasy Mini Kit (Qiagen, Inc., Valencia, CA, USA) according to manufacturer’s instruction as previously described [26,28,31]. Moreover, total RNA including miRNA was also extracted from serum samples using the miRNeasy Serum/Plasma Kit (Qiagen, Valencia, CA, USA). The concentrations and purity of the RNAs from whole blood and serum samples were assessed using a NanoDrop ND-1000 spectrophotometer (Thermo Fisher Scientific, Inc., Waltham, MA, USA). The concentrations of the whole blood RNAs ranged from 65–95 ng/µL, whereas the concentrations of the serum RNAs ranged from 5 to 30 ng/µL. Based on these findings, RNAs from whole blood samples were used in this study. All RNA samples were stored at −80 °C until further processing.

Reverse transcription (RT) was carried out using an Applied Biosystems TaqMan MicroRNA RT kit (Thermo Fisher Scientific). Briefly, total RNA (20 ng) was mixed with specific stem-loop RT primers (3 μL), 100 mM dNTPs (0.15 μL), 10X RT buffer (1.5 μL), 20 U/μL RNase inhibitor (0.19 μL), 50 U/mL MultiScribeTM Reverse Transcriptase (1 μL) and nuclease-free water (4.16 μL) to a final volume of 15 μL. The reaction mixtures were then incubated at 16 °C for 30 min, at 42 °C for 30 min and at 85 °C for 5 min and then held at 4 °C. cDNA samples were stored at −20 °C until analysis.

### 2.4. Reverse Transcription Quantitative Real-Time PCR

For reverse transcription, quantitative real time PCR (RT-qPCR) with TaqMan assays for Has-miR-375 (ID: 000564) and Has-miR-375 (ID: 002231) (Applied Biosystems; Thermo Fisher Scientific) were used. cDNA (2 μL) was used as a template in a 10 μL reaction mix containing 5 μL TaqMan 2X Universal PCR Master Mix II (Applied Biosystems; Thermo Fisher Scientific), 1 μL gene-specific primers and 2 μL nuclease-free water. The sequences of primers of miRNAs used for RT-qPCR are shown in Table 1.

RT-qPCR reactions were run with 7900HT real-time PCR system (Applied Biosystems; Thermo Fisher Scientific) 95 °C for 10 min, followed by 40 cycles of 95 °C for 15 s and 60 °C for 1 min. Duplicate measurements were obtained for each sample. The data were analyzed with SDS relative quantification software version 1.4 (Applied Biosystems; Thermo Fisher Scientific) with the automatic threshold cycle (Ct) setting for assigning baseline and threshold for Ct determination.

The small nuclear RNA, RNU6B, was used for normalization as its expression was demonstrated to be relatively abundant and constant across a wide range of human tissues and cell line types. It is regarded as one of the control genes with the least variability for miRNAs assays and has been widely used in different fields including diabetes research [28,31]. The relative expression of each individual miRNA was calculated using the 2−ΔΔCt method as previously described [26,28,31].

## 3. Statistical Analysis

Comparisons between the expression of miRNAs other clinical variables in cases and controls were analyzed by Student’s *t*-tests and among more than 2 groups were performed using one-way ANOVA (analysis of variance) as appropriate. Data were presented as the mean ± standard deviation (SD). The association of miRNAs with prediabetes and T2D was analyzed using multivariate logistic regression models with crude or adjusted odds ratio (OR). The linear correlation between miRNAs and other variables was determined using Pearson’s correlation coefficient. Receiver operating characteristic (ROC) analysis was used to assess the diagnostic accuracy of miRNAs and the area under the curve (AUC) was reported for each miRNA. Probability (*p*) value <0.05 was considered significant. All statistics were performed using SPSS software version 23 (IBM SPSS, Armonk, NY, USA).

## 4. Results

### 4.1. Basic Characteristics of the Study Subjects

In the present study, we included 30 prediabetes patients, 30 T2D patients and 30 non-diabetic healthy individuals. Table 2 summarizes the basic characteristics of the study subjects. The age of prediabetes patients ranged from 40 to 59 years (50 ± 5.8) and there were 19 males and 11 females, whereas the age of T2D patients ranged from 31 to 78 years (60 ± 12) and there were 12 males and 18 females. Whereas, the age of healthy subjects ranged from 47 to 66 years (56 ± 5.1) and there were 14 males and 16 females. The mean age differed significantly between T2D patients and healthy subjects and between prediabetes patients and T2D patients (*p* < 0.001). However, there was no significant difference in the mean age between prediabetes and healthy subjects (*p* > 0.05). As regards sex, there was no significant difference between the three groups (*p* > 0.05). In terms of glycemic status, prediabetes patients and T2D patients had significantly higher levels of FG, HbA1c and OGGT compared with the healthy subjects (*p* < 0.001). There were no significant differences between the study groups for a series of other parameters including BMI, hypertension, triglyceride, total cholesterol and HDL (*p* > 0.05). Whereas LDL differed significantly between subject groups and was higher in T2D patients than prediabetes patient and healthy subjects (*p* < 0.05).

### 4.2. Relative Expression of miR-375 and miR-9 in the Subject Groups

The expression of miR-375 and miR-9 normalization relative to the expression of RNU6B were determined by TaqMan-based RT-qPCR in peripheral whole blood of prediabetes patients, T2D patients and non-diabetic healthy control individuals.

**Figure 1:**
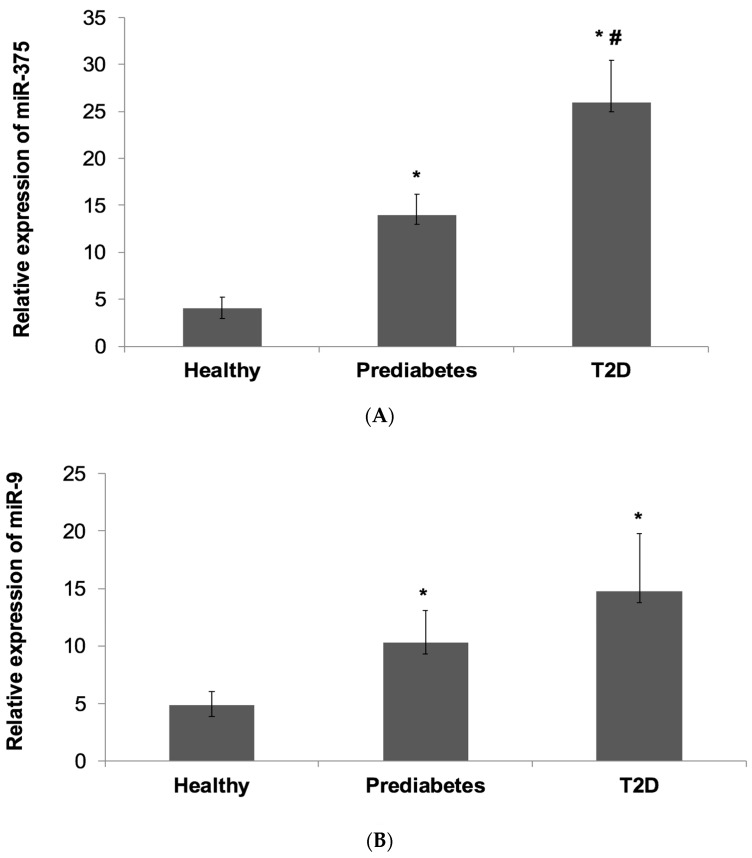
Relative Expression of miR-375 and miR-9 in the Subject Groups. The expression of miR-375 and miR-9 normalization relative to the expression of RNU6B were determined by TaqMan-based RT-qPCR in peripheral whole blood of prediabetes patients, T2D patients and non-diabetic healthy control individuals. (**A**): miR-375. (**B**): miR-9. Data is shown as mean ± SD. * *p* < 0.05 compared to healthy controls. ^#^ *p* < 0.05 compared to prediabetes.

Overall, the results showed that miR-375 and miR-9 were expressed at higher levels in prediabetes patients and progressively more enriched in T2D patients compared with non-diabetic healthy subjects (Figure 1).

miR-377 expression (Figure 1A) was significantly higher by 3-fold in prediabetes patients (13.6 ± 2.2) and was significantly increased by 5.9-fold in T2D patients (25.8 ± 4.5) compared with controls (4 ± 1.2) (*p* < 0.001). Notably, miR-375 expression was also significantly higher by 2-fold in T2D patients than prediabetes patients (*p* = 0.04).

As regards miR-9 expression (Figure 1B), it was significantly elevated by 2.1-fold in prediabetes patients (10.3 ± 2.8) and was significantly higher by 3-fold in T2D patients (14.8 ± 5) compared with controls (4.9 ± 1.2) (*p* < 0.001). However, although miR-9 expression was higher in T2D patients than in prediabetes patients, it was not significant (*p* > 0.05).

### 4.3. Multivariate Regression Analysis

To further assess the association between high expression miR-375 and miR-9 with prediabetes and T2D, multivariate logistic regression analysis was performed.

We found that both miR-375 and miR-9 were significantly associated with prediabetes and T2D when the group of non-diabetic healthy controls was treated as the reference category (Table 3A). For miR375, the odds ratio (OR) was 1.12 (95% confidence interval (CI): 1.022–1.168, *p* = 0.009) for prediabetes and the OR was 1.123 (95% CI: 1.049–1.201, *p* = 0.001) for T2D.

For miR-9, the OR was 1.11 (95% CI: 1.005–1.147, *p* = 0.035) for prediabetes and the OR was 1.080 (95% CI: 1.011–1.153, *p* = 0.022) for T2D. The association of the two miRNAs with the presence of prediabetes and T2D remained significant when different adjusted models were used in the analysis including adjustment such as age, sex, BMI, mean blood pressure, HbA1c, total cholesterol, triglyceride and LDL.

Next, we assessed the association between high expressions miR-375 and miR-9 with the development of T2D using the group of prediabetes patients as the reference category in multivariate logistic regression models (Table 3B).

The analysis revealed that miR-375 was significantly associated with T2D (OR: 1.1, 95% CI: 0.999–1.157, *p* = 0.05) and remained statistically significant following adjustment for age and sex and further for BMI mean blood pressure and additionally for total cholesterol, triglyceride and LDL (Table 3B). On the other hand, no significant association was found between miR-9 and T2D when the prediabetes group was treated as the reference category (OR: 1.006, 95% CI: 0.98–1.03, *p* = 0.64) and miR-9 also failed to show significance after multivariable adjustments.

### 4.4. Correlation between miR-375 and miR-9 with Glycemic Status and Other Clinical Variables

Pearson’s correlation coefficient analysis in prediabetes and T2D patients was performed to assess the relationship between high expression miR-375 and miR-9 with glycemic status and other clinical parameters.

The correlations between miR-375 and miR-9 with the glycemic status in prediabetes and T2D patients are demonstrated in Table 4A.

In the group of prediabetes patients, miR-375 displayed a significant positive correlation with FG (*p* = 0.006), HbA1c (*p* = 0.009) and OGGT (*p* = 0.002). Similarly, in the group of T2D patients, miR-375 showed a significant and positive correlation with FG (*p* = 0.02), HbA1c (*p* = 0.0024) and OGGT (*p* = 0.041).

As regards miR-9, in the group of prediabetes patients, a significant and positive correlation was observed with FG (*p* = 0.002), HbA1c (*p* = 0.002) and OGGT (*p* < 0.001). In the group of T2D patients, miR-9 was significantly and positively correlated with FG (*p* = 0.02) and HbA1c (*p* = 0.04) but showed a borderline significant correlation with OGGT (*p* = 0.07).

In addition, we further analyzed the correlation of high expressions of miR-375 and miR-9 with other clinical parameters including age, sex, BMI, hypertension, LDL, triglyceride and total cholesterol (Table 4B).

In the group of prediabetes patients, miR-375 was positively correlated with mean blood pressure and total cholesterol (*p* < 0.001) but negatively correlated with age, BMI, triglyceride and LDL (*p* < 0.001). In the group of T2D patients, miR-375 showed a positive correlation with age, BMI, mean blood pressure and total cholesterol (*p* < 0.001) and a negative correlation with triglyceride and LDL (*p* < 0.05).

For miR-9, it was positively correlated with age, BMI, mean blood pressure and total cholesterol (*p* < 0.05) and negatively correlated with triglyceride and LDL (*p* < 0.05) both in prediabetes and T2D patients.

### 4.5. Evaluation of the Diagnostic Values of Blood miR-375 and miR-9

To explore the possible roles of miR-375 and miR-9 as biomarkers for prediabetes and T2D, we evaluated their diagnostic values using the receiver operating characteristic (ROC) curve analysis.

**Figure 2:**
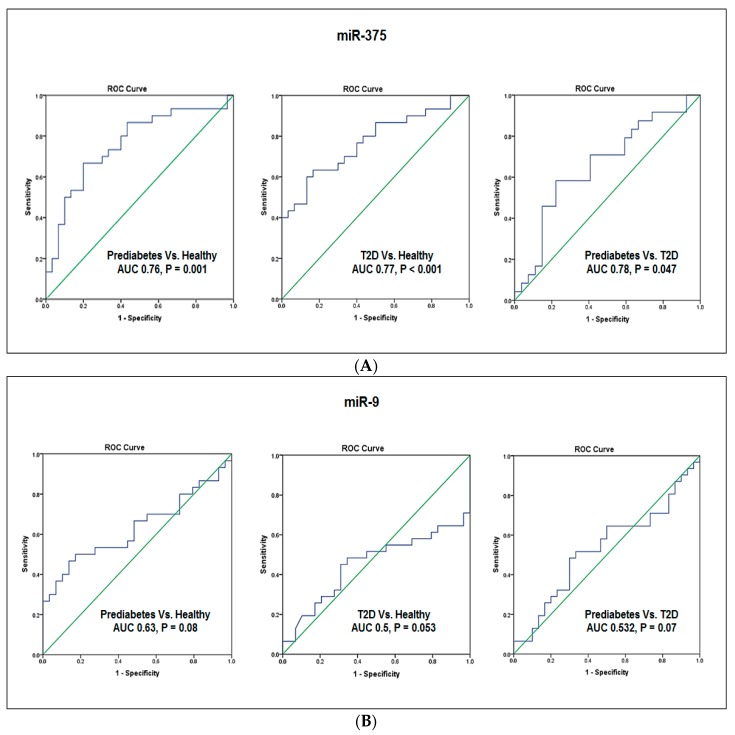

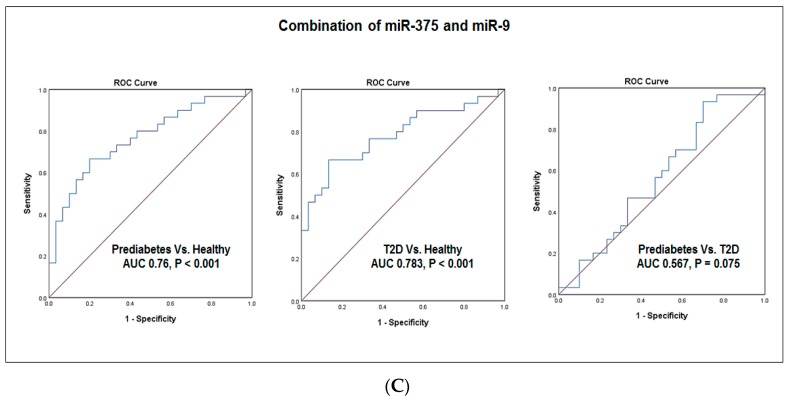
Receiver operating characteristic curve analysis. Receiver operating characteristic (ROC) curves were constructed to evaluate the diagnostic values of miR-375 and miR-9 as biomarkers for prediabetes and T2D. (**A**) ROC curves for miR-375. (**B**) ROC curves for miR-9. (**C**) ROC curves for combination of miR-375 with miR-9.

As shown in Figure 2A, the ROC analysis revealed a significant ability for miR-375 in discriminating between the subject groups. miR-375 discriminated prediabetes patients from healthy subjects with an area under the curve (AUC) of 0.76 (95% CI: 0.630–0.884, *p* = 0.001). miR-375 also discriminated T2D patients from healthy subjects with an AUC of 0.77 (95% CI: 0.65–0.89, *p* < 0.001). Interestingly, miR-375 differentiated between prediabetes patients and T2D patients with an AUC of 0.78 (95% CI: 0.504–0.81, *p* = 0.047).

On the other hand, miR-9 displayed a lower ability and a borderline significance in discriminating between the subject groups (Figure 2B). The AUC was 0.63 (95% CI: 0.485–0.777, *p* = 0.08) in discriminating prediabetes patients from healthy subjects and the AUC was 0.50 (95% CI: 0.301–0.604, *p* = 0.053) in discriminating T2D patients from healthy subjects, while the AUC was 0.532 (95% CI: 0.385–0.680, *p* = 0.07) in discriminating prediabetes patients from T2D patients. 

Notably, the combination of miR-375 with miR-9 (Figure 2C) enhanced the predictability to discriminate prediabetes patients from healthy subjects (AUC 0.767, 95% CI 0.64–0.88, *p* = 0.001) as well as T2D patients from healthy subjects (AUC 0.783, 95% CI 0.665–0.902, *p* = 0.001). However, the combined miR-375 with miR-9 did not significantly enhance the predictability to discriminate prediabetes patients from T2D patients (AUC 0.567, 95% CI 0.42–0.714, *p* = 0075).

## 5. Discussion

The increase in the incidence of prediabetes in parallel with the rapid increase of T2D prevalence worldwide [1,2] raises an urgent need to develop effective biomarkers for disease detection at an early stage to facilitate future preventive interventions and improve patient’s care [15,16]. microRNAs (miRNAs), a group of small non-coding RNA molecules, have been reported to regulate important cellular processes and are also implicated in many pathological conditions [19]. Being released by cells into the circulation and stalely detected in blood [24], miRNAs have shown promise as prognostic, diagnostic and predictive biomarkers [25,26,27]. Studies from our group and others have also demonstrated the stability and utility of peripheral blood miRNAs as biomarkers for prediabetes, T2D and other diseases [28,29].

Previous studies have demonstrated that aberrant expression of numerous miRNAs including miR-375 and miR-9 influence insulin signaling pathway, insulin resistance and play a major role in T2D [20]. Both miR-375 and miR-9 were previously reported as important regulators of insulin secretion and glucose homeostasis [22,23].

Therefore, in this study we examined the expression of the diabetes-related miR-375 and miR-9 by TaqMan-based RT-qPCR in peripheral blood of prediabetes patients, T2D patients and non-diabetic healthy individuals and evaluated their suitability as earlier blood biomarkers for detecting prediabetes and T2D.

We found that both miR-375 and miR-9 were expressed at higher levels in prediabetes patients and progressively more enriched in T2D patients as compared controls. We also observed that miR-375 expression was significantly higher in T2D patients than in prediabetes patients. On the other hand, although miR-9 expression was observed to be higher in T2D patients than prediabetes patents, it was not however significant.

Increased the expression of miR-375 and miR-9 in prediabetes and T2D patients are in agreement with a previous report by Kong et al. [32] showing increased miR-375 and miR-9 in sera from patients with prediabetes and T2D.

In our study, multivariate logistic regression analysis using crude an adjusted models revealed a direct association between miR-375 and miR-9 with the presence of prediabetes and T2D and this association remained significant following adjustment for age, sex, BMI, mean blood pressure, triglyceride, total cholesterol and LDL. These results may suggest an involvement of these two of miR-375 and miR-9 in the pathogenicity of T2D. Additionally, multivariate logistic regression analysis confirmed a significant association of miR-375 but not miR-9, with the development of T2D independently of age, sex, BMI, mean blood pressure, HbA1c, total cholesterol, triglyceride and LDL. These results indicate a possible link between miR-375 and the progression from prediabetes to T2D.

Both miR-375 and 9 are islet-specific microRNAs which expressed at high levels during human pancreatic islet development [21]. Poy et al. [22,33] have demonstrated that miR-375 is abundantly expressed in the pancreatic β–cells and essential for maintaining normal pancreatic cell mass. It functions as a negative regulate of glucose-stimulated insulin secretion through controlling the expression of myotrophin (Mtpn) and phosphoinositide-dependent protein kinase-1 (Pdk1) genes [22,34]. Overexpression of miR-375 suppresses insulin secretion, whereas its downregulation enhances insulin release [22]. Therefore, miR-375 is a key player in regulation of insulin release and glucose homeostasis [22].

miR-9 also participates in regulation of insulin secretion through the effect on insulin exocytosis from the pancreas [23]. Overexpression of miR-9 inversely correlates with glucose stimulated insulin release by directly targeting Onecut2 (OC2), the latter inhibits the expression of Granuphilin/Slp4. While, overexpression of OC2 suppresses Granuphilin transcription [35].

Impaired insulin secretion and action occur both in prediabetes and T2D patients [8,9]. These metabolic abnormalities may precede T2D for years [9,10] and a high percentage of prediabetes patients will progress to clinical T2D [9,10].

Based on the important regulatory roles of miR-375 and miR-9 in insulin secretion, upregulations of miR-375 and miR-9 in blood of prediabetes and T2D patients observed in the current study further support our earlier suggestion that these two miRNAs participation in the pathogenicity of T2D and miR-375 is associated with the T2D progression.

Several epidemiological studies have shown that factor like age, obesity, dyslipidemia, uncontrolled hyperglycemia, hypertension and family history contribute to increased risk of progression to T2D [36,37].

In our study, miR-375 and miR-9, which were significantly elevated in prediabetes and T2D patients, displayed positive correlations with FG and HbA1c both in the two groups of patients. Moreover, miR-375 and miR-9 were positively correlated with mean blood pressure and total cholesterol both in prediabetes and T2D patients and miR-9 was also positively correlated with age and BMI. These results may imply a link between the two miRNAs and the susceptibility to developing T2D.

Studies using animal models of diabetes have shown that increased circulating levels of miR-375 serve as useful predictor of diabetes and β–cell death [38,39].

In the present study, the ROC curve analysis revealed that miR-375 discriminated both prediabetes and T2D patients from healthy subjects and also differentiated between prediabetes patients from T2D patients with significant diagnostic ability.

Similar to our results, a previous study by Higuchi et al. [40], found a significant increase in the expression of miR-375 in T2D patients and suggested a possible use of this miRNA as a biomarker for T2D. Notably, our results extended these finding and showed that elevated miR-375 expression could be a useful biomarker not only for T2D but also to identify prediabetes patients who are at risk of developing T2D.

Contrary to miR-375, the ROC curve analysis in our study showed lower diagnostic value and borderline significance for miR-9 in discriminating between the three subject groups, suggesting that miR-9 is not a potentially good biomarker for prediabetes and T2D. Notably, the combination of miR-375 with miR-9 enhanced the predictability to discriminate prediabetes patients from healthy subjects as well as T2D patients from healthy subjects. However, the combined miR-375 with miR-9 did not significantly enhance the predictability to discriminate prediabetes patients from T2D patients. Despite these results, increased miR-9 expression in prediabetes and T2D patients and its correlation with T2D risk factors suggests an association with the susceptibility to T2D.

## 6. Conclusions

Our study demonstrated that blood miR-375 and miR-9 were expressed at higher levels in prediabetes and T2D patients compared with non-diabetic healthy subjects and progressively more enriched in T2D patients. Both miRNAs were directly associated with the presence of prediabetes and T2D independently of known risk factors to T2D and miR-375 was independently associated with the development of T2D. miR-375 and miR-9 were positively correlated with the glycemic status and other T2D risk factors, implying a link between the two miRNAs and the susceptibility to developing T2D. Our study also showed a high diagnostic ability for miR-375 but not miR-9, to distinguish overall patients from control and prediabetes from T2D patients, whereas, the combination of miR-375 with miR-9 -375 showed a good diagnostic ability to discriminate overall patients from controls, indicating that miR-375 alone or in combination with miR-9 could serve as biomarkers for early detection of prediabetes and T2D. Further validation studies in larger sample size are still needed before the clinical application miR-375 as a biomarker for prediabetes and T2D.

## Figures and Tables

**Table 1:** Sequences of primers of miRNAs used RT-qPCR.

	Forward Primer 5′-3′	Reverse Primer 5′-3′
miR-375	GAGCATTTTGTTCGTTCGGC	AGTGCAGGGTCCGAGG
miR-9	GCCCGCTCTTTGGTTATCTAG	CCAGTGCAGGGTCCGAGGT
RNU6B	GCTTCGGCAGCACATATACTAAAAT	CGCTTCACGAATTTGCGTGTCAT

**Table 2:** Basic characteristics of the study participants.

Characteristics	Prediabetes	T2D	Controls
Number of subjects	30	30	30
Age (years)	50 ± 5.8 ^##^	60 ± 12 **	56 ± 5.1
Sex (M/F)	(19/11)	(12/18)	(14/16)
FG (mmol/L)	6.4 ± 5.8 **^##^	8.6 ± 13.6 **	4.3 ± 0.6
HbA1c (%)	6.7 ± 0.5 **^##^	8.68 ± 2.6 **	5.03 ± 0.7
2 h OGTT (mmol/L)	8.71 ± 0.69 **^##^	13.72 ± 2.03 **	6.00 ± 0.75
Diabetes duration (years)	-	15 ± 4.4	-
BMI (Kg/m^2^)	25.0 ± 4.7	25.7 ± 5.2	24.2 ± 4.6
Mean blood pressure (mmHg)	89 ± 5.4	87.5 ± 5.3	86.9 ± 4.0
Triglyceride (mmol/L)	1.53 ± 0.56	1.54 ± 0.5	1.60 ± 0.6
Total cholesterol (mmol/L)	4.1 ± 1.3	4.54 ± 1.1	4.27 ± 0.6
LDL (mmol/L)	2.08 ± 0.9	2.36 ± 1.1 *	2.14 ± 0.8
HDL (mmol/L)	1.32 ± 0.4	1.28 ± 0.2	1.34 ± 0.3

The values are represented by either numbers, percentages (%) for categorical data, mean ± standard deviation for parametrically distributed data, or median (interquartile range) for non-parametrically distributed data. T2D, type 2 diabetes mellitus; BMI, body mass index; FG, fasting glucose; HbA1c, glycated hemoglobin; OGTT, oral glucose tolerance test; HDL, high density lipoprotein; LDL, low density lipoprotein. * *p* < 0.05, ** *p* < 0.001 compared with the non-diabetic subjects; ^#^ *p* < 0.05, ^##^ *p* < 0.001 compared with T2D patients.

**Table 3:** (**A**) Multivariate regression analysis of miRNAs for the presence of prediabetes and T2D. (**B**) Multivariate regression analysis of miRNAs for the development of T2D.

	Prediabetes			T2D		
(**A**)						
**miR-375**						
Models	OR	95% CI	*p* value	OR	95% CI	*p* value
Model ^1^	1.12	1.022–1.168	0.009	1.123	1.049–1.201	0.001
Model ^2^	1.11	1.023–1.174	0.009	1.134	1.056–1.219	0.001
Model ^3^	1.12	1.012–1.26	0.023	1.125	1.045–1.212	0.022
Model ^4^	1.13	1.012–1.168	0.022	1.126	1.045–1.214	0.002
**miR-9**						
Model ^1^	1.11	1.005–1.147	0.035	1.080	1.011–1.153	0.022
Model ^2^	1.12	1.010–1.157	0.024	1.082	1.012–1.157	0.021
Model ^3^	1.1	1.002–1.150	0.044	1.078	1.007–1.154	0.032
Model ^4^	1.1	1.006–1.161	0.035	1.081	1.007–1.159	0.031
(**B**)						
**miR-375**						
Models	-			OR	95% CI	*p* value
Model ^1^	-			1.12	0.999–1.157	0.05
Model ^2^	-			1.141	1.001–1.528	0.001
Model ^3^	-			1.143	1.001–1.483	0.044
Model ^4^	-			1.151	1.006–1.197	0.025
**miR-9**						
Model ^1^	-			1.006	0.982–1.030	0.64
Model ^2^	-			1.001	0.976–1.026	0.954
Model ^3^	-			1.003	0.975–1.030	0.85
Model ^4^	-			0.972	0.916–1.031	0.33

T2D, type 2 diabetes; OR, odds ratio; CI, confidence interval. (**A**) The reference category was the non-diabetic healthy control group. Model ^1^: crude; Model ^2^: included age and sex. Model ^3^: included age, sex, BMI and mean blood pressure. Model ^4^: included age, sex, BMI, mean blood pressure, total cholesterol, triglyceride and LDL. (**B**) The reference category was the prediabetes group. Model ^1^: crude. Model ^2^: included age and sex. Model ^3^: included age, sex, BMI, mean blood pressure and HbA1c. Model ^4^: included age, sex, BMI, mean blood pressure, HbA1c, total cholesterol, triglyceride and LDL.

**Table 4:** (**A**) Correlation between miR-375 and miR-9 with glycemic status. (**B**) Correlation between miR-375 and miR-9 with other clinical variables.

Variables	miR-375				miR-9			
	Prediabetes		T2D		Prediabetes		T2D	
**(A)**	*r*	*p* value	*r*	*p* value	*r*	*p* value	*r*	*p* value
FG	0.30	0.006	0.20	0.020	0.20	0.002	0.30	0.020
HbA1c	0.30	0.009	0.10	0.024	0.13	0.002	0.41	0.04
OGGT	0.35	0.002	0.40	0.041	0.44	<0.001	0.42	0.07
**(B)**	*r*	*p* value	*r*	*p* value	*r*	*p* value	*r*	*p* value
Age	−0.12	0.001	0.02	0.001	0.02	0.001	0.05	0.001
Sex	-0.12	0.535	−0.20	0.369	0.21	0.269	0.30	0.143
Diabetes duration	-	-	0.13	0.507	-	-	−0.13	0.483
BMI	−0.01	0.001	0.35	0.001	0.40	0.001	0.1	0.034
Mean blood pressure	0.28	0.001	0.11	0.001	0.11	0.001	0.12	0.001
Triglyceride	−0.30	0.001	−0.20	0.006	−0.20	0.006	−0.11	0.010
Total cholesterol	0.02	0.001	0.41	0.047	0.41	0.047	0.01	0.043
LDL	−0.26	0.001	−0.33	0.015	−0.33	0.015	−0.10	0.014

(**A**) T2D, type 2 diabetes mellitus; *r*, Pearson’s correlation coefficient, FG, fasting glucose; HbA1c, glycated hemoglobin; OGTT, oral glucose tolerance test. (**B**) T2D, type 2 diabetes; *r*, Pearson’s correlation coefficient; BMI, body mass index; LDL, low density lipoprotein.
